# Self-organization of network dynamics into local quantized states

**DOI:** 10.1038/srep21360

**Published:** 2016-02-17

**Authors:** Christos Nicolaides, Ruben Juanes, Luis Cueto-Felgueroso

**Affiliations:** 1 Sloan School of Management, Massachusetts Institute of Technology, Cambridge, MA, USA; 2 Department of Civil and Environmental Engineering, Massachusetts Institute of Technology, Cambridge, MA, USA; 3 Department of Hydraulics, Energy and Environment, Technical University of Madrid, Spain

## Abstract

Self-organization and pattern formation in network-organized systems emerges from the collective activation and interaction of many interconnected units. A striking feature of these non-equilibrium structures is that they are often localized and robust: only a small subset of the nodes, or cell assembly, is activated. Understanding the role of cell assemblies as basic functional units in neural networks and socio-technical systems emerges as a fundamental challenge in network theory. A key open question is how these elementary building blocks emerge, and how they operate, linking structure and function in complex networks. Here we show that a network analogue of the Swift-Hohenberg continuum model—a minimal-ingredients model of nodal activation and interaction within a complex network—is able to produce a complex suite of localized patterns. Hence, the spontaneous formation of robust operational cell assemblies in complex networks can be explained as the result of self-organization, even in the absence of synaptic reinforcements.

Pattern formation in reaction-diffusion systems^1^,^2^ has emerged as a mathematical paradigm to understand the connection between pattern and process in natural and sociotechnical systems^3^. The basic mechanisms of pattern formation by local self-activation and lateral inhibition, or short-range positive feedback and long-range negative feedback^4^,^5^ are ubiquitous in ecological and biological spatial systems, from morphogenesis and developmental biology^1^,^6^ to adaptive strategies in living organisms^7^,^8^ and spatial heterogeneity in predator-prey systems^9^. Heterogeneity and patchiness associated with Turing patterns in vegetation dynamics have been proposed as a connection between pattern and process in ecosystems^10^,^11^, suggesting a link between spatial vegetation patterns and vulnerability to catastrophic shifts in water-stressed ecosystems^12^,^13^,^14^.

The theory of non-equilibrium self-organization and Turing patterns has been recently extended to network-organized natural and socio-technical systems^15^,^16^,^17^,^18^, including complex topological structures such as multiplex^19^,^20^, directed^21^ and cartesian product networks^22^. Self-organization is rapidly emerging as a central paradigm to understand neural computation^23^,^24^,^25^. The dynamics of neuron activation, and the emergence of collective processing and activation in the brain, are often conceptualized as dynamical processes in network theory^26^,^27^,^28^. Self-organized activation has been shown to emerge spontaneously from the heterogenous interaction among neurons^25^, and is often described as pattern formation in two-population networks^29^,^30^,^31^,^32^. Localization of neural activation patterns is a conceptually challenging feature in neuroscience. Cell assemblies, or small subsets of neurons that fire synchronously, are the functional unit of the cerebral cortex in the Hebbian theory of mental representation and learning^33^,^34^,^35^,^36^,^37^. Associative learning forms the basis of our current understanding of the structure and function of neural systems^38^,^39^,^40^. It is also the modeling paradigm for information-processing artificial neural networks^41^,^42^,^43^. The emergence of cell assemblies in complex neural networks is a fascinating example of pattern formation arising from the collective dynamics of interconnected units^25^,^44^. Understanding the mechanisms leading to pattern localization remains a long-standing problem in neuroscience^34^,^44^,^45^,^46^,^47^,^48^.

Here we show that simple mechanisms of nodal interaction in heterogeneous networks allow for the emergence of robust local activation patterns through self-organization. The simplicity and robustness of the proposed single-species pattern-forming mechanisms suggest that analogous dynamics may explain localized patterns of activity emerging in many network-organized natural and socio-technical systems. We demonstrate that robust local, quantized activation structures emerge in the dynamics of network-organized systems, even for relatively simple dynamics. We propose a minimal-ingredients, phenomenological model of nodal excitation and interaction within a network with heterogeneous connectivity. Our goal is to demonstrate that a simple combination of local excitation of individual units, combined with generic excitatory/inhibitory interactions between connected units, leads to self-organization, and can explain the spontaneous formation of cell assemblies without the need for synaptic plasticity or reinforcement. Our model can be understood as a network analogue of the Swift-Hohenberg continuum model^49^,^50^,^51^, and is able to produce a complex suite of localized patterns. The requirements are minimal and general: simple local dynamics based on canonical activation potentials, and interactions between nodes that induce short-range anti correlation and long-range correlation in activation. Because of their robustness and localization, self-organized structures may provide an encoding mechanism for information processing and computation in neural networks.

## Model of Network Dynamics and Stability Analysis

**Figure 1:**
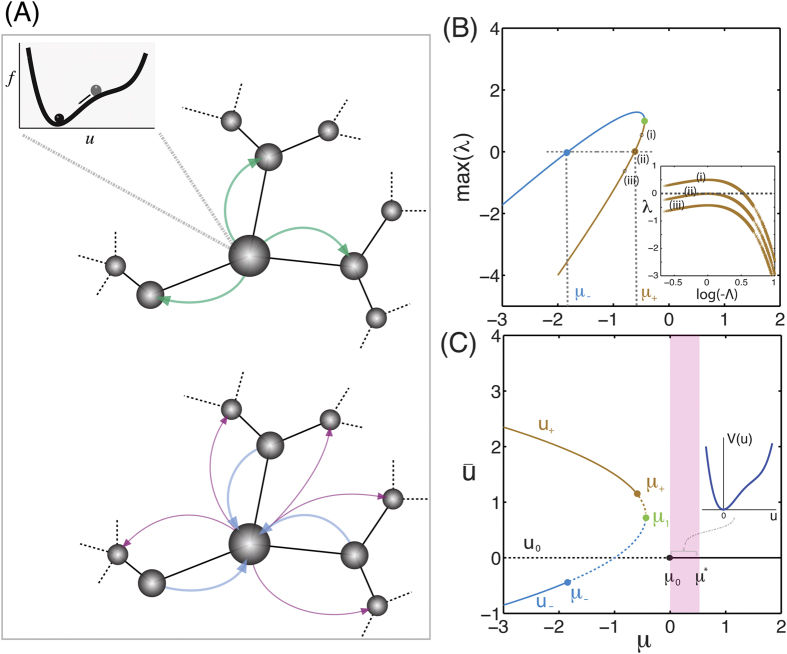
(**A**) *Pictorial illustration of our dynamical model of network interactions.* Locally, the nodal activation state is driven by the dynamic forcing term *f* (*u*, *μ*). In the inset we show the potential landscape—minus the integral of *f* with respect to u, which exhibits a single well (at *u* = 0) with an inflection point, a necessary condition for localized patterns to exist. Nodes interact in the network through diffusion-like exchanges via the links connecting them. The network Laplacian operator, *L*^(2)^, represents short range diffusion of the species in the system (top). The network bi-Laplacian operator, *L*^(4)^, induces short range anti-correlation with the nearest-neighbors, and long-range correlation with the second-nearest neighbors (bottom). (**B**,**C**) *Linear stability analysis of the flat stationary solutions of our model.* (**B**) The maximum value of the growth rate *λ* as a function of the bifurcation parameter *μ* for the two flat stationary states *u*_+_ (brown) and *u*_−_ (blue) on a Barabási-Albert network model with mean degree 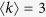 and size *N* = 2000. When the maximum value of *λ* is negative, the state is stable with respect to small non uniform perturbation. (Inset) The growth rate *λ* as a function of the Laplacian eigenvalue Λ (Eq. (5)) for three different values of the bifurcation parameter *μ* as indicated in the main diagram for the flat stationary solution *u*_−_. (**C**) The flat stationary solutions *u*_0_ and *u*_±_ as a function of *μ* on the same network. Solid (dotted) lines represent stability (instability) with respect to small non-uniform perturbations. The labelled bifurcation points are *μ*_0_ = 0, *μ*_1_ = −0.44 and *μ*_+_ = −0.62 and *μ*_−_ = −1.82. The pink shaded region is where we observe localized self-organization patterns with respect to the trivial solution *u*_0_. For values of *μ* outside that region we get either global activation patterns (for *μ* < *μ*_0_) or any perturbation relaxes back to the flat stationary solution (for *μ* > μ^*^).

We restrict our analysis to the simplified case of symmetric networks, but our main results can be generalized to other network topologies, including directed^21^ and multiplex^19^,^20^ networks. A node’s state of activation, measured through a potential-like variable *u*, is driven by local excitation dynamics and by the interaction with other nodes in the network via exchanges through the links connecting them. In dimensionless quantities, the proposed excitation-inhibition model for the evolution of potential, *u*_*i*_, in each node 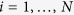, is given by the model
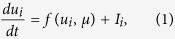
where *f *(*u*_*i*_, *μ*) is a dynamic forcing term, representing a double well potential, and *μ* is a bifurcation parameter that will be used to establish the conditions for stability and localization of the response patterns (Fig. 1A). The currents, *I*_*i*_, represent the excitatory/inhibitory interactions among nodes in the network. The structure of these nodal interactions is one of the key pattern forming mechanisms in the present model. We consider short-range anti-correlation, and higher-order, longer-range dissipative interactions. This two-level interaction structure, which induces anti correlation in the short range (nearest-neighbors, or first-order connectivity), and long-range correlation (second-nearest neighbors, or second-order connectivity) is represented in Fig. 1A. Mathematically, we express the integration of synaptic contributions as
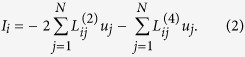


The structure of the above nodal interactions turns the dynamics (1)–(2) into a network anologue of the Swift-Hohenberg continuum model,
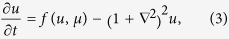
which is a paradigm for pattern-forming systems^49^,^50^,^51^,^52^,^53^. The simplest form for the interaction matrices representing these correlation/anti-correlation effects (while ensuring that the interaction fluxes conserve mass or charge) is based on network representation of Laplacian and bi-Laplacian operators, ***L***^(2)^ and ***L***^(4)^, respectively. The network Laplacian, ***L***^(2)^, is a real, symmetric and negative semi-definite *N* × *N* matrix, whose elements are given by 
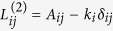
16, where *A*_*ij*_ is the adjacency matrix of the network, 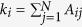 is the degree (connectivity) of node *i* and *δ*_*ij*_ is the Kronecker delta. A diffusive, Fickian-type flux of the activation potential *u* to node *i* is expressed as 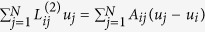 (see Fig. 1A–top). Plain waves and wavenumbers on a network topology are represented by the eigenvectors 
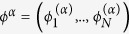
 and the eigenvalues Λ_*α*_ and of the Laplacian matrix, which are determined by the equation 
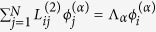
, with 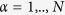
2. All eigenvalues are real and non-positive and the eigenvectors are orthonormalized as 
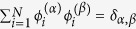
, where 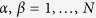. The elements of the bi-Laplacian matrix of a network can be expressed as 
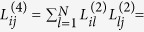

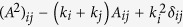
 where the 
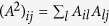
 matrix has information about second order nodal connectivity and takes nonzero values if node *i* is two jumps away from node *j*. The operation 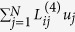 models negative diffusion (inhibition) from the first neighbors of node *i* and at the same time diffusion from its two-jump neighborhood (see Fig. 1A–bottom). The bi-Laplacian, ***L***^(4)^, has the same eigenvectors as ***L***^(2)^ (i.e. *ϕ*^*α*^) and its eigenvalues are the square of those of ***L***^(2)^, 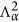.

To understand the properties and pattern-forming mechanisms in our model, we first investigate the stability of flat states of the dynamical system (2):
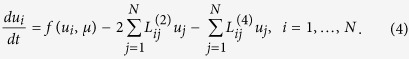


Flat, stationary solutions 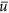 of Eq. (4) satisfy *f *(*u*_*i*_, *μ*) = 0, where the nodal state of activation is equal for all nodes in the network, 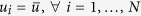. For 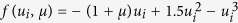, there are three uniform solution branches given by *u*_0_ = 0 and 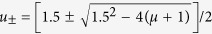. It is well known in one and two dimensional continuum spaces that these uniform states can become unstable and a wealth of self-organized patterns can arise^49^,^50^,^51^,^52^,^53^. In a linear stability analysis, the stability of flat stationary solutions to small perturbations is determined by the eigenvalues of the Laplacian and bi-Laplacian matrices. Introducing small perturbations, *δu*_*i*_, to the uniform state 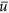, 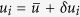, the linearized version of Eq. (4) takes the form 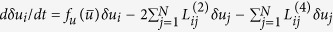, where 
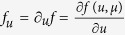
. After expanding the perturbation *δu*_*i*_ over the set of the Laplacian eigenvectors, 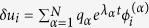, where *q*_*α*_ is the expansion coefficient, the linearized equation is transformed into a set of *N* independent linear equations for the different normal modes:
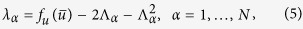
where Λ_*α*_ are the eigenvalues of the Laplacian matrix. The *α*-mode is unstable when Re *λ*_*α*_ is positive. Instability occurs when one of the modes (the critical mode) begins to grow. At the instability threshold, Re *λ*_*α*_ = 0 for some *α*_*c*_ and Re *λ* < 0 for all other modes. In Fig. 1B,C we summarize the linear stability analysis of the flat states of our model on a scale-free network constructed using the Barabási-Albert model (BA) of network growth and preferential attachment^54^. We find that, indeed, there is a large parameter range for which the resting potential is stable. As we demonstrate below, in the stable regime, input stimuli may trigger localized patterns of activation.

## Localized Patterns

**Figure 2:**
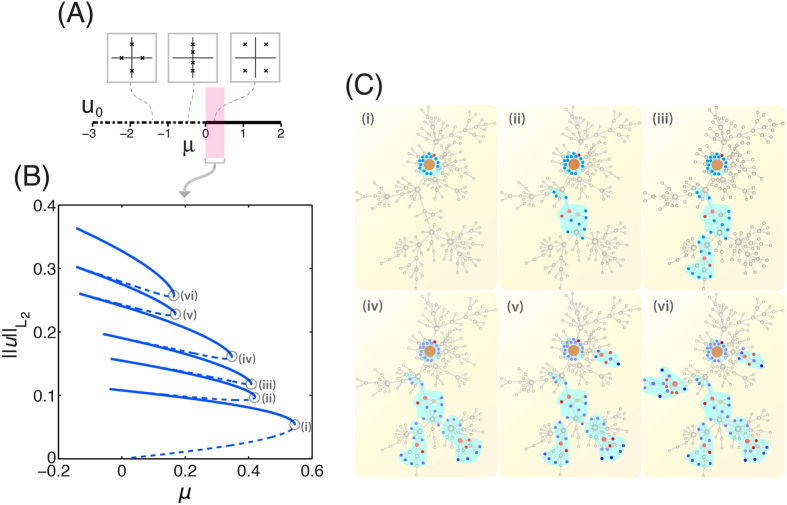
Localized self-organized quantized patterns. (**A**) Stability of the trivial flat stationary state of our model with respect to the values of the bifurcation parameter, *μ*. For positive values of *μ* the trivial stationary solution is stable with respect to uniform small random perturbations (solid line) while for negative values of *μ* this state becomes unstable (dotted line). Also shown in the insets are the topological eigenvalues of the trivial state as we tune the bifurcation parameter. The behavior of eigenvalues in the neighborhood of *μ* = 0 indicates the possibility for localized patterns in the neighborhood of small positive values of *μ* (pink shaded region). (**B**) A single branch of the bifurcation diagram in a Barabási-Albert network model of size *N* = 200 with mean degree equal to 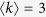 and minimum degree equal to 1. Solid (dotted) lines represent stable (unstable) localized solutions. (**C**) Visualization of the localized patterns corresponding to the states indicated on the bifurcation diagram (**B**). Gray-colored nodes are non-active (*u* = 0), red-colored nodes are active with *u* > 0 and blue-colored nodes are active with *u* < 0. The size of the node is proportional to its eigenvalue centrality.

Localized activation patterns are possible due to the particular structure of the model, with short- and long-range nodal interactions. Mathematically, the localized states are homoclinic orbits in the network space around the base resting state, 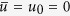. The existence of these homoclinic orbits can be studied using the technology developed for the linear stability analysis. Since homoclinic orbits leave the flat state as we approach a small neighborhood (cluster) of the network, the fixed point must have both stable and unstable eigenvalues. We linearize Eq. (4) around *u* = 0 and write 
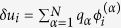
, 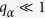, arriving to the relation 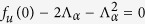. Since the Laplacian eigenvalues Λ_*α*_ are real and non-positive values, we can write them in the form 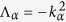. If *μ* > 0 the topological eigenvalues of *u* = 0 form a complex quartet 
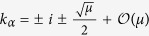
. For *μ* = 0 they collide pairwise on the imaginary axis, and for *μ* < 0 they split and remain on the imaginary axis 
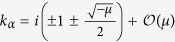
. For *μ* = −1 two of the topological eigenvalues collide at the origin and for *μ* < −1 they move onto the real axis. These results are summarized in Fig. 2A. The topological eigenvalues in the neighborhood of *μ* = 0 are characteristics of the reversible 1 : 1 resonance bifurcation. Theory shows that under certain conditions the hyperbolic regime contains a large variety of topologically localized states^51^.

To understand the onset of localized patterns for different model parameters and input stimuli, we construct the bifurcation diagram of the resting state, as a function of the total potential energy of the stimulus and bifurcation parameter *μ*, in the vicinity of 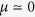
51,52. A single bifurcation branch—constructed using a pseudo-arclength continuation method^55^—has a characteristic “snaking” structure of localized states with varying activation energy 
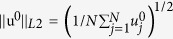
 (Fig. 2B). As the system jumps from one steady state branch to the next one, a new neighborhood in the network is being activated. Figure 2C visualizes the different steady localized states of the six different branches as they are spotted in the diagram of Fig. 2B. The response of the system is *quantized*: the transition from one pattern of activation to another one is discontinuous as we vary the activation energy 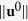, or the parameter *μ* (Fig. 2B). These jumps in activation energy correspond to the addition of neighbor nodes to the cluster (Fig. 2C).

**Figure 3:**
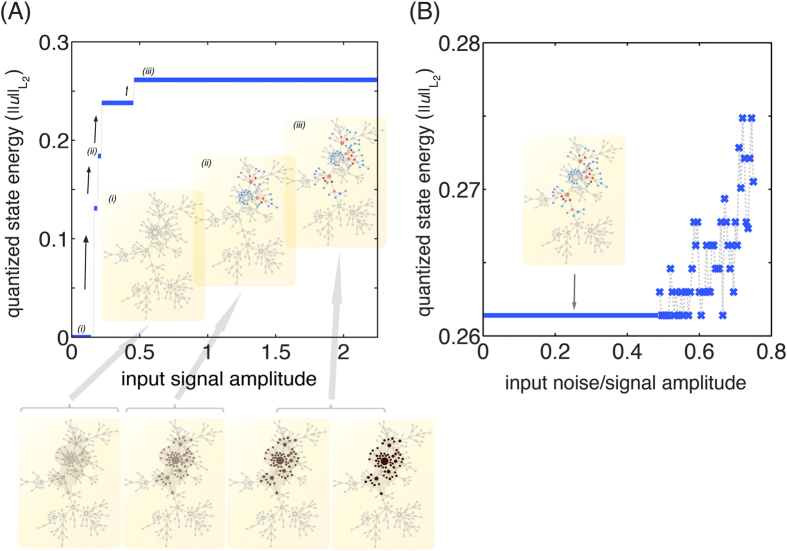
Robustness of quantized patterns. (**A**) Energy of the resulting quantized state with respect to the input signal amplitude 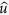 at the nearest and next-nearest neighbors of the best connected node in the system. When the amplitude is very small, the initial perturbation relaxes back to the trivial solution and no quantized state is formed (*i*). As the amplitude of the input signal is increased, fragile quantized states are formed (*ii*). When the amplitude of the input signal is larger than a threshold value, a very robust quantized state is formed (*iii*). Further increases in the input signal amplitude lead to the same quantized state. (insets) Visualization of the input signal in our network topology (the amplitude increases from left to right) as well as the resulting equilibrium state. (**B**) The energy of the resulting quantized state with respect to the ratio between the signal amplitude and the noise amplitude. Starting from the step-like input signal that gives the robust quantized state, we add random noise at the already perturbed neighborhood and we compute the energy of the resulting quantized state over 100 realizations. We use a Barabási-Albert scale-free network of size *N* = 200 and mean degree 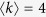.

The discontinuous—quantized—nature of the network response leads to *robustness* in the local, final equilibrium patterns with respect to the input signal amplitude. To gain insight into the robustness of the localized patterns of activation, we performed a synthetic test in which we initially stimulate a specific neighborhood in the network, where we set 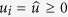 (i.e. a step-like function signal in network topology) and let the system evolve to equilibrium without decay. We gradually increase the amplitude 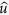 of the initial signal, and record the final energy values of the equilibrium, localized states. For small amplitudes the perturbation relaxes back to the resting state, and no activation pattern is elicited. There is a threshold in the energy of the input stimulus beyond which robust quantized states are formed. The states are robust in the sense that further increments in the input signal amplitude do not change the final equilibrium pattern (Fig. 3A).

The self-organized local structures are also robust with respect to random noise in the initial stimulus. We perform Monte Carlo simulations that probe the impact of the noise-to-signal ratio on the energy of the emerging quantized state. We have confirmed that the presence of small-amplitude noise has no effect on the equilibrium states of nodal activity. As can be expected, we do observe a departure from the energy of the base equilibrium state when the noise-to-signal ratio is sufficiently large, thereby masking the base stimulus altogether (Fig. 3B).

## Mean-Field Approximation of the Global Activation Patterns

Our model predicts a range of parameter values where localized states disappear, and are replaced by *global activation patterns*. Mathematically, global patterns are possible when the non-active stationary solution is perturbed outside the parameter region of localized patterns (*μ* < 0). These—global—Turing patterns^1^,^16^ can be understood and modeled using the Mean-Field Approximation (MFA), a method that segregates nodes according to their degree and has been successfully used to approximate a wide variety of dynamical processes in heterogeneous networks, like epidemic spreading^56^,^57^,^58^, activator-inhibitor models^16^ and voter models^59^.

This theory allow us to reduce the problem to a single equation for the membrane potential for all the nodes in the system. Since in our model both the degree and two-jump degree play important role in the formation of patterns, we use a MFA where we assume that all the nodes with the same degree and two-jump degree behave in the same way. We start by writing Eq. (3) in the form
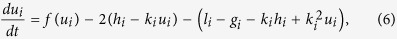
where the local fields felt by each node, 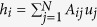, 
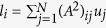
 and 
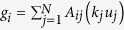
 are introduced. These local fields are then approximated as 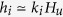, 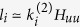 and 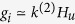, where 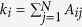 is the degree and 
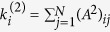
 is the number of secondary connections of node *i* (two-jump degree). The global mean fields are defined by 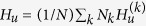 where 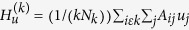 and 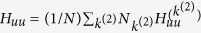, where 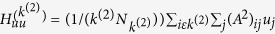. Here, *N*_*k*_ is the number of nodes with degree *k*, 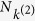 is the number of nodes with *k*^(2)^ number of two-jump neighbors and 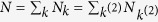 is the size of the network. In the above expressions, with 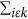 we denote the sum over the nodes with degree *k* and with 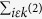 the sum over the nodes with two-jump nodal connectivity *k*^2^.

With this approximation, the individual model equation on each node interacts only with the global mean fields *H*_*u*_ and *H*_*uu*_ and its dynamics is described by:
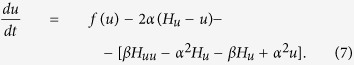


Since all nodes obey the same equation, we have dropped the index *i* and introduced the parameters *α*(*i*) = *k*_*i*_ and 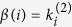. The activation potential depends now on the global fields *H*_*u*_ and *H*_*uu*_ as well as on the parameter compination (*α*, *β*), i.e. 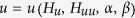. If the global mean fields *H*_*u*_ and *H*_*uu*_ are given, the combination (*α*, *β*) plays the role of a bifurcation parameter that controls the dynamics of each node in the system. The time independent version of above mean field equation can be written as a third degree algebraic equation that we solve *N* times for the *N* nodes in the system. For each node *i*, we get three solutions 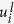, 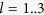 that can be stable or unstable depending on the sign (negative or positive respectively) of the operator 
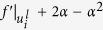
.

**Figure 4:**
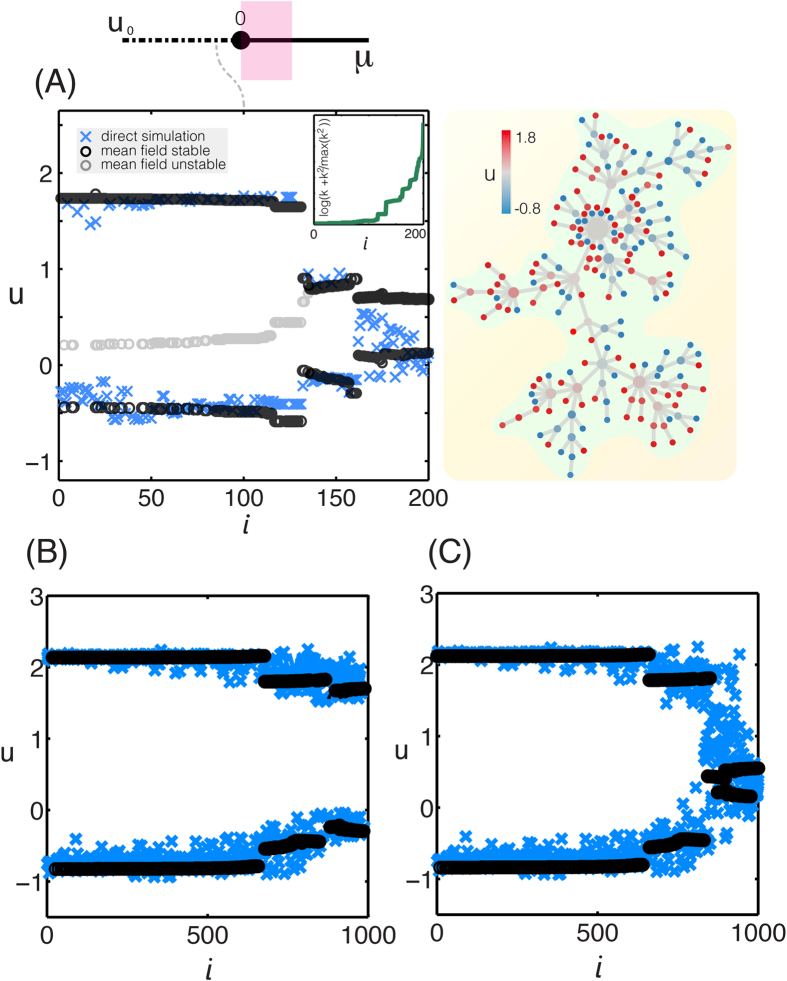
Global Turing patterns. Global patterns are possible when the non-active stationary solution is perturbed outside the parameter region of localized patterns (*μ* < 0). The initial exponential growth of the perturbation is followed by a nonlinear process leading to the formation of stationary Turing patterns. (**A**) (left) The activation profile as a function of the node index *i* of a global stationary Turing pattern from direct simulation (blue crosses) is compared with the mean-field bifurcation diagram. Black curves indicate stable branches while grey curves correspond to unstable branches of a single activator–inhibitor system coupled to the computed global mean fields. We sort the node index *i* in increasing connectivity *k*. Nodes with the same degree are sorted with increasing two-jump connectivity *k*^(2)^ (see Inset). We use the same Barabási-Albert network model as in Fig. 2 and we set the bifurcation parameter equal to *μ* = −1/4. We have confirmed that similar results hold for larger network sizes. (right) Visualization of the global activity pattern on the network topology. (**B**) The activation profile as a function of the node index *i* of global stationary Turing patterns from direct simulation for bifurcation parameter *μ* = −0.25 on an Erdös-Rényi random network with size *N* = 1000 and mean degree 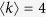 (blue curve) along with the stable branch of the mean field approximation (black curve). (**C**) The activation profile as a function of the node index *i* of global stationary Turing patterns from direct simulation on a Barabási-Albert scale free network with the same mean degree and the same number of node as the Erdös-Rényi of B. We sort the node index in increasing connectivity *k* and two-jump connectivity *k*^(2)^.

After tuning the bifurcation parameter *μ* to a negative value, we can compute the global Turing pattern from direct numerical simulations and determined the global mean fields *H*_*u*_ and *H*_*uu*_. Each node *i* in the network is characterized by its degree and second nodal connectivity, so that it possesses a certain parameter combination, (*α*, *β*). Substituting these computed global mean fields as well as the values of *α* and *β* into equation (7), bifurcation diagrams of a single node can be obtained and projected onto the Turing pattern. In Fig. 4A we show for our “toy network model” that the stable brunches of the nodal bifurcation diagrams calculated using the MFA fit very well the computed Turing pattern. We further assess the dependence of the network topology on the global pattern formation and we find that when the degree distribution is narrower compared to a scale-free network, the distribution of the (*α*, *β*) is more homogeneous and therefore the stationary Turing patterns look smoother (Fig. 4B,C). Therefore, global network Turing patterns are essentially explained by the bifurcation diagrams of individual nodes coupled to the global mean fields, with the coupling strength determined by their degree and two-jump connectivity.

## Conclusions

Our results suggest a new mechanism for the formation of localized nodal assemblies in networks, arising from long-range—second neighbor—interactions. Rather than relying on reinforcing mechanisms—synaptic plasticity, we show that localized, robust nodal assemblies are possible due to self-organization. The emergence of localized activation patterns derived from the simple and general functional structure of our proposed conceptual model: local dynamics based on activation potentials, and interactions between nodes that induce short-range anticorrelation and long-range correlation in node-to-node exchanges. The proposed system is a network analogue of the Swift-Hohenberg continuum model, and is able to produce a complex suite of robust, localized patterns. These self-organized, local structures can provide robust functional units to understand natural and technical network-organized processes.

## Additional Information

**How to cite this article**: Nicolaides, C. *et al*. Self-organization of network dynamics into local quantized states. *Sci. Rep.* **6**, 21360; doi: 10.1038/srep21360 (2016).
